# Frequency, Risk Factors, and Methods of Deliberate Self-Harm Observed in Medical Students and Doctors in Pakistan

**DOI:** 10.7759/cureus.70068

**Published:** 2024-09-24

**Authors:** Sundus Mona, Muhammad Bilal Rehman, Abdul Ahad, Amjad Farooq, Ramlah Naz, Samina Rehman, Aftab Alam Tanoli, Zahid Bashir

**Affiliations:** 1 Department of Forensic Medicine and Toxicology, Shalamar Medical and Dental College, Lahore, PAK; 2 Department of Community Medicine, Shalamar Medical and Dental College, Lahore, PAK; 3 Department of Medicine, Shalamar Medical and Dental College, Lahore, PAK; 4 Medicine, Shalamar Hospital, Lahore, PAK; 5 Department of Forensic Medicine and Toxicology, Dow International Medical College, Karachi, PAK; 6 Department of Forensic Medicine, Bolan Medical College, Quetta, PAK; 7 Department of Forensic Medicine and Toxicology, Women Medical College, Abbottabad, PAK

**Keywords:** attempted suicide, borderline personality disorder (bpd), deliberate self-harm (dsh), mental health disorders, non-suicidal self-inflicting injury (nssi), self-esteem, self-inflicted harm, substance abuse

## Abstract

Objective: To determine the frequency of deliberate self-harm (DSH), risk factors leading to DSH, and the methods adopted by medical students and young doctors to execute DSH.

Method: A cross-sectional study was conducted on medical college students and young doctors aged 18-26 years. After approval by the parent institute’s ethical board, different medical students and doctors from Pakistani medical colleges of both male and female populations were recruited through non-probability sampling. Responses were collected from one to two medical colleges from each province.

Results: A high number of 490 (60.9%) out of 805 participants reported a history of at least one form of self-harm. Forty-four participants (0.05%) reported having attempted suicide in the past. The mean scores for the self-harm inventory scale were 2.51±3.25, the self-esteem scale 26.51±4.43, and the social support scale 23.1±6.96. Smoking, recreational drug use, History of mental illness, and family history of mental illness were significantly associated with increased odds of self-harm in medical students. On adjusted binary logistic regression, female gender, harmonious relationship with parents, satisfaction with the result, social support, and higher self-esteem were protective against deliberate self-harm.

Conclusion: DSH is a critical issue among medical students and is becoming prevalent. Higher odds of DSH are associated with smoking and recreational drug use. Higher self-esteem and better social support are protective against DSH.

## Introduction

Deliberate self-harm is a specific non-fatal behaviour where a person hurts themself intentionally by cutting skin, taking drugs, or committing harmful acts with the sole purpose of harming their own body [1]. According to an estimate, in Pakistan alone, there are 130,000 to 270,000 cases of deliberate self-harm every year. According to WHO, every suicide is preceded by 10-20 acts of deliberate self-harm, to the minimum [2]. According to the recent statistics of WHO reports on self-harm or suicide that were estimated in the year 2012, 13,377 cases of suicides/self-harm in Pakistan were observed, including 7085 females and 6021 males, with a rate of 7.5/100,000. There has been a rise of 2.6% in cases since the year 2000. According to these statistics, there could be nearly 130,000 to 270,000 acts of DSH in Pakistan per annum [2]. Deliberate self-harm is under-researched and less reported because it is penalized in Pakistan as per Section 325 of the Pakistan Penal Code [3]. As suicide and deliberate self-harm are a social stigma, such cases are either hidden or left unaccounted for at legal, cultural, and religious levels [4].

Owing to the numerous forms of stress during academic years due to complicated and demanding course schedules and high expectations, the prevalence of self-harm in medical students goes up to more than 10% lifetime risk [5-7]. As medical graduation courses are challenging courses, medical students are more likely to suffer from social and environmental stress that leads to the tendency of deliberate self-harm and suicide ideation, even attempts [8]. Furthermore, the risk factors for developing stress-related issues are related to curriculum, accommodation, a network of social support, and academic versus peer pressures. These interpersonal factors lead to personal or social isolation and competitive academic anxiety at the earliest educational time. Other endemic issues directly linked to medical studies are simulation and cadaver sessions and eye witnessing pain and suffering in the hospitals. If students are vulnerable already, high expectations from teachers and families exacerbate psychological pressures to succeed [9].

The objectives of this study are to determine the frequency of DSH among medical students and young doctors aged 18-26 years, identify the risk factors leading to DSH in this population, including personal, familial, and environmental influences, examine the methods employed by these individuals to engage in DSH, investigate protective factors, such as self-esteem and social support, that may reduce the likelihood of DSH.

Deliberate self-harm should be studied in the context of personal attributes like self-esteem, social support, and family factors, and medical students are a population that needs to be checked more carefully to understand the dilemma of deliberate self-harm in Pakistan.

## Materials and methods

After obtaining ethical approval from the parent institute's review board, participants were selected using a non-probability convenience sampling method. This method, chosen for its practicality and efficiency, allowed for the inclusion of a diverse range of medical students and young doctors aged 18-26 from various medical colleges across Pakistan. A total of 45 medical colleges were included from all provinces and the state of Azad Jammu and Kashmir, with each institution contributing a different number of participants.

The inclusion criteria included medical students and young doctors currently enrolled in a medical program, aged 18-26 years, willing to participate in the study voluntarily. Exclusion criteria included incomplete responses, participants not meeting the age requirement, and students enrolled in non-medical curriculums.

To ensure a high response rate and efficient data collection, a self-administered questionnaire was distributed using Google Forms, a method known for its user-friendly interface and accessibility. This approach was well-received by participants and included three standardized scales, which were free to use and widely accepted. 

The first scale was Rosenberg's Self-esteem scale [10], a widely used psychological research tool measuring global self-worth through 10 items. Participants rated statements such as "I feel that I am a person of worth" and "I feel I do not have much to be proud of" on a 4-point Likert scale, ranging from "strongly agree" to "strongly disagree."

The second scale was Sarason's Social support questionnaire [11], which evaluates perceived social support. Participants indicated the number of people they could rely on for help and their satisfaction with this support through questions like "How many people do you know you can count on for support?" and "How satisfied are you with the support you receive?"

The third scale was Sansone and Wiederman's Self-harm inventory [12], a 22-item tool assessing self-harming behaviors. Participants responded with "yes" or "no" to questions like "Have you ever intentionally cut yourself?" or "Have you ever purposely hurt yourself to feel emotional relief?" A "yes" response to any item was classified as indicative of deliberate self-harm (DSH).

These scales assessed key psychological dimensions, including self-esteem, perceived social support, and self-harm behaviors. The calculated sample size for this study was 620, based on a 95% confidence level, a 3% margin of error, and an expected self-harm prevalence of 17.3% among medical students. The sample size was determined using the WHO formula: n= z²(pq)/d².

Eight hundred sixty-five responses were collected. Incomplete responses and responses from non-medical institutes were excluded from the analysis, leaving us with 805 responses. The data collected was exported to SPSS software, Version 26 (IBM Corp., Armonk, NY) for statistical analysis. Frequencies were expressed in percentages and bar charts. Quantitative variables were defined as Mean and Standard Deviation. The Chi-square test of independence was used to study the association of deliberate self-harm with different variables. Binary logistic regression was used for bivariate and multivariate analysis.

## Results

Out of 865 responses, 60 responses were excluded based on exclusion criteria. Of the 805 participants, the mean age was 21.5 ± 2.04 years, who participated in this study, 68.8% were females, and more than half (59.6%) were Punjabi ethnicity. This is relevant as 66 out of 123 medical colleges, representing 54% of the total medical colleges, are located in Punjab. This fact helps to eliminate provincial bias, ensuring that the sample is representative of the broader population, given the distribution of medical colleges across regions.

**Figure 1:**
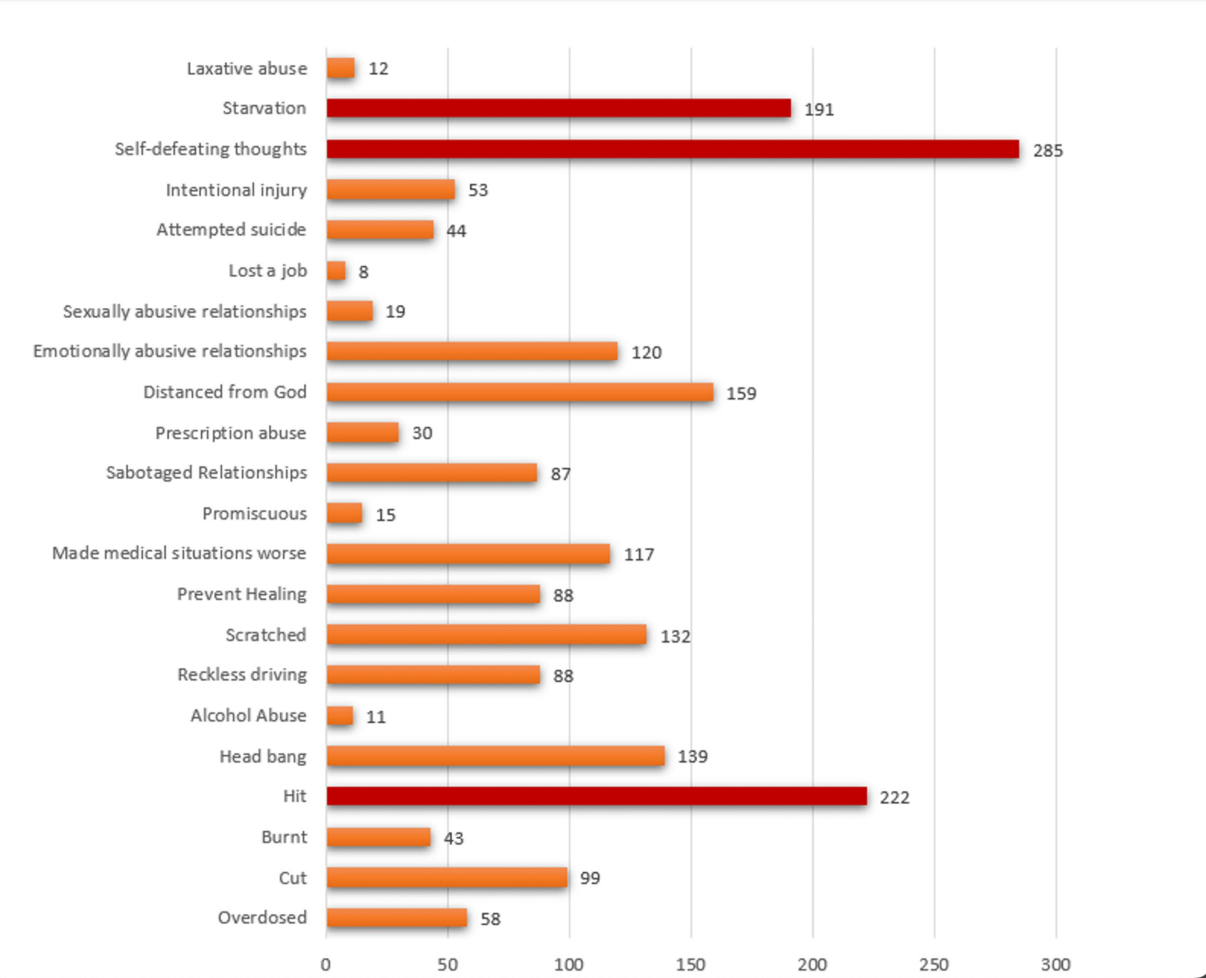
Frequencies of various forms of deliberate self-harm in medical students and doctors in Pakistan

More than three-quarters (77.9%) of the students were satisfied with the results of their last exams. Almost 86% of participants reported a harmonious relationship with their parents. A high percentage of 60.9% (n=490) participants reported a history of at least one form of self-harm throughout their life, whereas 21.5% (n=173) were found to have borderline personality disorder. A total of 44 participants (0.05%) reported a history of suicide attempts (Figure 1).

The mean scores for the Self-harm inventory scale were 2.51±3.25, the Self-esteem scale 26.51±4.43, and the Social support scale 23.1±6.96 (Table 1).

**Table 1:** Frequency and association of various factors with any form of deliberate self-harm in medical students and doctors in Pakistan

Variables	Frequencies		Chi-Square Values	p-value
	Category	n (%)		
Gender	Males	251 (31.2%)	8.066	0.005
	Females	554 (68.8%)		
Ethnicity	Punjabi	480 (59.6%)	8.33	0.214
	Pashtun	116 (14.4%)		
	Balochi	62 (7.7%)		
	Sindhi	37 (4.6%)		
	Kashmiri	26 (3.2%)		
	Afghani	3 (0.4%)		
	Other	81 (10.1%)		
Year of Study	1^st^ Year	126 (15.7%)	9.47	0.149
	2^nd^ Year	145 (18%)		
	3^rd^ Year	313 (38.9%)		
	4^th^ Year	120 (14.9 %)		
	Final Year	67 (8.3 %)		
	House Officer	14 (1.7%)		
	Medical officer	20 (2.5%)		
Satisfaction with the Result of Last Year	Yes	627 (77.9%)	14.197	<0.001
	No	178 (22.1%)		
Harmonious Relationship with Parents	Yes	694 (86.2%)	20.158	<0.001
	No	111 (13.8%)		
Parents education	No Formal Education	47 (5.8%)	0.266	0.966
	Undergraduates	137 (17%)		
	Graduation	329 (40.9%)		
	Post-graduation	292 (36.3%)		
Parents employment status	Employed	522 (64.8%)	1.65	0.438
	Unemployed	246 (30.6%)		
	Deceased	37 (4.6%)		
Number of siblings	0	21 (2.6%)	2.53	0.639
	1	74 (9.2%)		
	2	170 (21.1%)		
	3	222 (27.6%)		
	>3	318 (39.5%)		
Living condition	Home	340 (42.2%)	0.88	0.64
	Hostel	449 (55.8%)		
	With relatives	16 (2%)		
Difficult basic needs	Easy	531 (66%)	4.10	0.250
	Difficult	52 (6.5%)		
	Relatively hard	70 (8.7%)		
	Manage somehow	152 (18.9%)		
Smoking	Yes	49 (6.1%)	4.695	0.030
	No	756 (93.9%)		
Recreational Drugs Use	Yes	15 (1.8 %)	4.271	0.039
	No	790 ( 98.1%)		
History of Physical Illness	Yes	57 (7%)	5.467	0.19
	No	748 (92.9%)		
History of Mental Illness	Yes	102 (12.6%)	36.723	<0.001
	No	703 (87.3%)		
Family History of Mental Illness	Yes	87 (10.8%)	15.16	<0.001
	No	718 (89.1%)		
Social Support	Mean	23.14± 6.96	84.464	<0.001
	Range	0-36		
Rosenberg Self Esteem	Value	86.60	86.60	<0.001

On Chi-square analysis and binary logistic regression, female gender was significantly associated with a self-reported history of any form of self-harm (p=0.005, odds ratio (OR) for females 0.635 CI 95% 0.464-0.870). Satisfaction with the results of the last exam was associated with a lesser likelihood of self-harm (p<0.001, OR 0.498 (0.345-0.719). A harmonious relationship with parents, better social support, and higher self-esteem were negatively associated with a history of self-harm. Whereas tobacco smoking, recreational drug use, and mental illness of self or family were positively associated with self-harm. No significant association of self-harm was found with Ethnicity (p=0.214), Year of study (p=0.159), Living situation (p=0.643), Parents’ education (p=0.996), and Parents’ employment status (p=0.438).

Variables with a p-value on Chi-square analysis less than 0.1 were included for adjusted binary logistic regression. Gender, Harmonious relationship with parents, History of personal and family mental illness, and self-esteem were independently associated with Deliberate self-harm (Table 2).

**Table 2:** Bivariate and adjusted logistic regression for factors associated with deliberate self-harm in medical students and doctors in Pakistan

Variables	Bivariate Binary logistic regression		Adjusted Binary logistic regression	
	Odds Ratio (OR)	p-value (p)	Odds Ratio (OR)	p-value (p)
Gender	0.635(0.464-0.870)	5	0.54(0.38-0.77)	1
Satisfaction with the Result of Last Year	0.498(0.345-0.719)	<0.001	0.70(0.46-1.03)	0.07
Harmonious Relationship with Parents	0.338 (0.207-0.552)	<0.001	0.46(0.27-0.77)	3
Smoking	2.06 (1.05-4.01)	30	1.03 (0.48-2.19)	0.94
Recreational Drugs Use	4.265 (0.956-19.030)	39	2.33 (0.47-11.63)	0.30
History of Mental Illness	5.681(3.055-10.567)	<0.001	3.66(1.90-7.07)	<0.001
Family History of Mental Illness	2.873(1.656-4.985)	<0.001	2.13(1.18-3.83)	0.01
Social Support	0.935(0.915-0.956)	<0.001	0.97(0.95-1.00)	47
Rosenberg Self Esteem	0.88(0.85-0.91)	<0.001	0.91(0.87-0.95)	<0.001

## Discussion

Deliberate self-harm (DSH) in youngsters leads to suicidal tendencies, ideation, and attempts, and medical students are no exception. DSH is related to social and environmental risk factors and can easily be linked to mental health issues and substance dependence. This cross-sectional study was conducted exclusively on medical students studying in different medical colleges in all four provinces of Pakistan.

DSH in youngsters has been increasing in prevalence in recent years [13,14]. According to an international study comprising a cohort of 10 countries, the percentage of youth suffering from depression and self-harm increased to as high as 57% in 2020 [15]. These results were close to our study that showed self-harm prevalence to be 60.9% for at least one form of self-harm in medical undergraduates. Similarly, a systematic review from 2008 reported that several studies from Pakistan found an association between young age and deliberate self-harm (DSH). Our study showed 0.05% of previous suicide attempts, while another study from undergraduate medical students showed (3%-15%) suicidal ideation [16]. 

DSH was found to be less likely in female medical students in our study. Similarly, a cross-sectional study from India reported that male medical students scored higher on risk-taking and self-harm inventory as compared to female students [17]. Multicentered research in Islamabad and Rawalpindi with purposive sampling of medical students at risk of DSH found no significant difference among male and female university students on scores of Inventory of Statement about Self-Injury (ISAS), a 39 Item questionnaire with good reliability [18]. A systematic review of 23 researchers from Pakistan published in 2008 reported that females were more at risk of deliberate self-harm than males [13]. This difference can be because of the different populations in the studies or a shift in pattern over time.

The most prevalent form of DSH, according to our study, was self-defeating thoughts, self-hitting, and starvation (Figure 1). Limited studies from Pakistan have reported standard methods of NSSI; however, the most common forms of suicide reported from Pakistan include hanging, poisons, and firearms.

Factors positively associated with self-harm in medical undergraduates included tobacco smoking, drug abuse, history of mental and physical illness, and family history of mental illness (Table 2). These findings corresponded with previous studies showing both substance use and borderline personality disorder lifetime rates from 45.5% and 86.6% co-occurring with Non-Suicidal Self Injury. Patients with mental health illnesses and drug-associated problems frequently showed up in emergency rooms with OD, intoxication, and self-inflicted or accidental trauma [19].

Protective factors towards deliberate self-harm in medical students identified by our study included satisfaction with academic performance, good relationship with parents, Higher self-esteem, and higher social support. Parental support has been associated with a low risk of suicide ideation and attempts [20]. The negative relationship between higher self-esteem and NSSI has been proven both in adults and adolescents [21,22].

The high percentage of DSH in this study may be due to several factors. It reflects lifetime history, not current prevalence, and the 22-item Self-harm inventory includes a broad range of behaviors, from physical harm to emotional and spiritual distress, such as self-defeating thoughts and distancing from God. Emotional and spiritual self-harm were more commonly reported than physical acts like cutting. Additionally, convenience sampling among medical students may have led to higher participation from those with mental health issues, potentially influencing the results.

Our study's limitations included the cross-sectional design and non-probability sampling. Additionally, although established scales such as Rosenberg’s self-esteem scale, Sarason’s social support questionnaire, and the Self-harm inventory by Sansone and Wiederman were used, these scales do not determine the timeline of behaviors and instead ask about the lifetime history of self-harm, which limits the ability to assess recent or time-bound incidents.

However, our study's strengths included nationwide data collection and the use of standardized tools. Further, prospective studies should be conducted on medical undergraduates from Pakistan to recognize and address this growing issue. Interventions like self-esteem counseling and parental training for better relationships can potentially reduce the incidence of self-harm in medical students.

## Conclusions

Deliberate self-harm (DSH) is alarmingly common among medical students and young doctors, likely driven by the pressures of their demanding academic and work environments. Key protective factors, such as higher self-esteem and positive relationships with parents, were found to reduce the likelihood of self-harm, while risk factors like mental health issues, tobacco use, and recreational drug use increased vulnerability. Gender and satisfaction with academic performance also played a role in DSH risk.

These findings highlight the urgent need for greater awareness and intervention strategies to address self-harm and mental health challenges in this high-stress population, focusing on building emotional resilience and supportive environments.
